# The Global Network Maternal Newborn Health Registry: a multi-country, community-based registry of pregnancy outcomes

**DOI:** 10.1186/s12978-020-01020-8

**Published:** 2020-11-30

**Authors:** Elizabeth M. McClure, Ana L. Garces, Patricia L. Hibberd, Janet L. Moore, Shivaprasad S. Goudar, Sarah Saleem, Fabian Esamai, Archana Patel, Elwyn Chomba, Adrien Lokangaka, Antoinette Tshefu, Rashidul Haque, Carl L. Bose, Edward A. Liechty, Nancy F. Krebs, Richard J. Derman, Waldemar A. Carlo, William Petri, Marion Koso-Thomas, Robert L. Goldenberg

**Affiliations:** 1 Social, Statistical and Environmental Health Sciences, RTI International, 3040 Cornwallis Rd., Durham, NC 27709 USA; 2 Instituto de Nutrición de Centroamérica y Panamá, Guatemala City, Guatemala; 3 School of Public Health, Boston University, Boston, MA USA; 4 KLE Academy Higher Education and Research, J N Medical College, Belagavi, Karnataka India; 5 Aga Khan University, Karachi, Pakistan; 6 Moi University School of Medicine, Eldoret, Kenya; 7 Lata Medical Research Foundation, Nagpur, India; 8 University Teaching Hospital, Lusaka, Zambia; 9 International Centre for Diarrhoeal Disease Research, Bangladesh (icddr,b), Dhaka, Bangladesh; 10 University of North Carolina At Chapel Hill, Chapel Hill, NC USA; 11 Indiana School of Medicine, University of Indiana, Indianapolis, IN USA; 12 University of Colorado School of Medicine, Denver, CO USA; 13 Thomas Jefferson University, Philadelphia, USA; 14 University of Alabama At Birmingham, Birmingham, AL USA; 15 University of Virginia, Charlottesville, VA USA; 16 Eunice Kennedy Shriver National Institute of Child Health and Human Development, Bethesda, MD USA; 17 Department of Obstetrics and Gynecology, Columbia University School of Medicine, New York, NY USA

**Keywords:** Registry, Perinatal mortality, Neonatal mortality, Stillbirth, Maternal mortality, Global network

## Abstract

**Background:**

The Global Network for Women's and Children’s Health Research (Global Network) conducts clinical trials in resource-limited countries through partnerships among U.S. investigators, international investigators based in in low and middle-income countries (LMICs) and a central data coordinating center. The Global Network’s objectives include evaluating low-cost, sustainable interventions to improve women’s and children’s health in LMICs. Accurate reporting of births, stillbirths, neonatal deaths, maternal mortality, and measures of obstetric and neonatal care is critical to determine strategies for improving pregnancy outcomes. In response to this need, the Global Network developed the Maternal Newborn Health Registry (MNHR), a prospective, population-based registry of pregnant women, fetuses and neonates receiving care in defined catchment areas at the Global Network sites. This publication describes the MNHR, including participating sites, data management and quality and changes over time.

**Methods:**

Pregnant women who reside in or receive healthcare in select communities are enrolled in the MNHR of the Global Network. For each woman and her offspring, sociodemographic, health care, and the major outcomes through 42-days post-delivery are recorded. Study visits occur at enrollment during pregnancy, at delivery and at 42 days postpartum.

**Results:**

From 2010 through 2018, the Global Network MNHR sites were located in Guatemala, Belagavi and Nagpur, India, Pakistan, Democratic Republic of Congo, Kenya, and Zambia. During this period at these sites, 579,140 pregnant women were consented and enrolled in the MNHR, nearly 99% of all eligible women. Delivery data were collected for 99% of enrolled women and 42-day follow-up data for 99% of those delivered. In this supplement, the trends over time and assessment of differences across geographic regions are analyzed in a series of 18 manuscripts utilizing the MNHR data.

**Conclusions:**

Improving maternal, fetal and newborn health in countries with poor outcomes requires an understanding of the characteristics of the population, quality of health care and outcomes. Because the worst pregnancy outcomes typically occur in countries with limited health registration systems and vital records, alternative registration systems may prove to be highly valuable in providing data. The MNHR, an international, multicenter, population-based registry, assesses pregnancy outcomes over time in support of efforts to develop improved perinatal healthcare in resource-limited areas.

*Trial Registration* The Maternal Newborn Health Registry is registered at Clinicaltrials.gov (ID# NCT01073475). Registered February 23, 2019. https://clinicaltrials.gov/ct2/show/NCT01073475

## Introduction

Accurate data are critical to understanding the progress in reducing maternal, fetal and neonatal mortality over time and across countries as well as to measure the impact of interventions designed to reduce mortality [1]. Historically, pregnancies are not accurately registered in many low-resource settings and maternal, fetal and newborn deaths have not been counted. To estimate pregnancy outcomes in these settings, periodic household surveillance studies have been supported, yet these have well-documented limitations including recall bias [2]. On the other hand, many of the research studies addressing pregnancy outcomes have been done in hospitals, which provides an incomplete picture of outcomes in low-resource settings, especially where many births occur at home [3]. These data on pregnancy and birth outcomes are critical to understand and ultimately improve pregnancy outcomes in low-resource settings.

The Global Network’s Maternal Newborn Health Registry (MNHR) is a prospective, population-based registry implemented in the catchment areas of low-resource countries [4–11]. The MNHR began in 2008 in research sites in Argentina, Guatemala, India (2 sites), Pakistan, Kenya and Zambia. In subsequent funding cycles, sites in the Democratic Republic of the Congo (DRC) and most recently, Bangladesh were added. The site in Argentina left the Global Network because it became ineligible, due to its rising economic status.

The MNHR provides data to assess the trends in pregnancy outcomes over time and between regions in order to provide population-level data for defined geographic areas. For example, we have observed that while there have been modest declines in the major outcomes of interest, including stillbirth, 28-day neonatal mortality and maternal mortality, these rates remain substantially higher than observed in high-income countries [10]. Of the participating sites, Pakistan continues to have the highest rates of adverse outcomes followed by the DRC site [11]. Additionally, the MNHR provides data on birth outcomes for individual trials as well as epidemiologic studies to inform further research. Following the FIRST BREATH trial of newborn resuscitation [12], the MNHR informed results of the many of the major Global Network trials, including the Antenatal Corticosteroids Trial, the FIRST LOOK ultrasound and preconception nutrition trials and more recently, the trial of low-dose aspirin to reduce risk of preterm birth [13–17]. Thus, because accurate data are needed not only to assess trends but also to evaluate the impact of interventions, the MNHR has been an important source of accurate pregnancy-related information to inform both local and global estimates of maternal and newborn mortality.

The objective of this publication was to describe the MNHR, including an overview of the study sites, its organization and management, methods of data collection and the changes over time. Additional descriptive data for the sites and major pregnancy outcomes including stillbirth, and maternal and neonatal mortality from the MNHR are in the manuscripts in this supplement [18–20].

## Methods

### MNHR organization and management

The MNHR is conducted within the *Eunice Kennedy Shriver* National Institute of Child Health and Human Development (NICHD) Global Network for Women’s and Children’s Health Research (Global Network). The Global Network has been funded by NICHD since 2001 as a cooperative agreement, comprising grantees representing a partnership between U.S. academic institutions with institutions based in a low or low-middle income country [6]. The Global Network conducts both interventional as well as observational studies addressing pregnancy and child outcomes.

The MNHR Steering Committee, consisting of investigators from each site and a representative from the NICHD and the Data Coordination Center (DCC), guides the general conduct of the MNHR. The Steering Committee oversees the use of MNHR data, data analyses and publications. Over the study period, the sites participating in MNHR have evolved. In 2008, the MNHR was initiated at the sites based in Argentina, Guatemala, Belagavi and Nagpur, India, Pakistan, Kenya, and Zambia. In 2013, following the NICHD’s re-competition of the Global Network, the site in Argentina, which no longer met the World Bank criteria for low or low/middle income, was replaced by a site based in the DRC. The Bangladesh site joined the Global Network and began MNHR data collection in 2019 (Table 1).

**Table 1:** Sites of the global network for women’s and children’s health research

Location of site	In-country institution	US institution	Senior foreign principal investigator	US principal investigator
African region				
Democratic Republic of Congo	Kinshasa School of Public Health, Kinshasa	University of North Carolina-Chapel Hill	Antoinette Tshefu	Carl Bose
Kafue and Chongwe Provence, Zambia	University Teaching Hospital, Lusaka	University of Alabama at Birmingham	Elwyn Chomba	Waldemar Carlo
Busia, Bungoma and Kakamega Counties, Kenya	Moi University, Eldoret, Kenya	Indiana University School of Medicine	Fabian Esamai	Edward Liechty
Asia region				
Belagavi, Karnataka, India	Jawaharlal Nehru Medical College, Belagavi	Thomas Jefferson University, Philadelphia PA	Shivaprasad Goudar	Richard Derman
Thatta, Pakistan	Aga Khan University, Karachi	Columbia University	Sarah Saleem	Robert Goldenberg
Nagpur, India	Lata Medical Research Foundation	Boston University	Archana Patel	Patricia Hibberd
Dhaka, Bangladesh	icddr,b	University of Virginia	Rashidul Haque	William Petri
Central America				
Chimaltenango, Guatemala	Instituto de Nutrición de Centroamérica y Panamá (INCAP), Guatemala City	University of Colorado School of Medicine	Lester Figueroa	Nancy Krebs
Data Coordinating Center	RTI International, Durham, NC		Elizabeth McClure	

At each site, the MNHR is overseen by the senior site investigator and study coordinator. One or more field supervisors at each site then manage the daily field activities for the MNHR. Each cluster employs a research administrator (RA) who is responsible for data collection, entry, and transmission of data to the DCC. Typically, the RAs are healthcare providers within the community. The RAs work closely with the existing healthcare service providers to help ensure that data describing pregnancies are comprehensive and accurate, as described elsewhere [7]. This study enrollment has been facilitated through community leaders (e.g. village elders and ministry of health officials) and the RAs may access medical charts of participating health facilities (e.g. delivery logs). In addition to field staff, each site employs a data manager to ensure accurate data entry, identifying and resolving edits to improve the data quality.

### Global Network MNHR sites

**Fig. 1:**
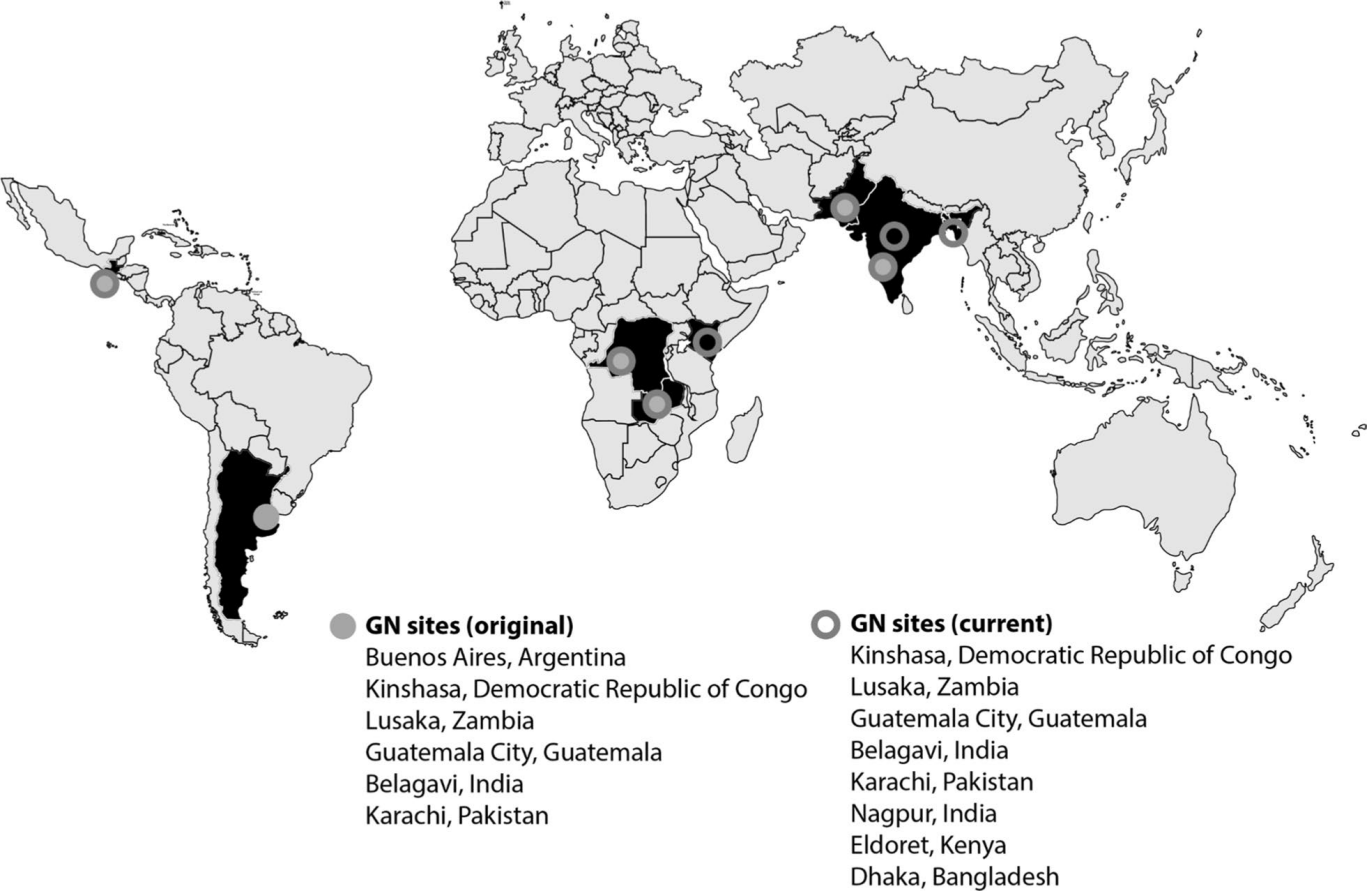
Map of Global Network research sites. Note: Original includes those which initiated the study in 2008; current includes those sites active in MNHR as of 2019

The Global Network sites have been funded through five-year grants and thus the sites have changed since the Global Network’s initiation in 2001 (Fig. 1). This section describes the sites that were active as of 2019.

### South Asian sites

#### Bangladesh

The site comprises two sub-districts of Tangail district, located about 60 miles northwest of the capital, Dhaka, where the research coordinating center is based. Each cluster has a primary health care center, which is staffed by a Family Welfare Visitor and a Sub-Assistant Community Medical Officer. The clusters also have community clinics, the lowest tier heath facility, two in each cluster, each of which is staffed by a community health care provider. In addition to the public health facilities, the sub-districts also have private clinics/hospitals, which provide inpatient maternal and child-care services including cesarean delivery. The private clinics have general practitioners and some specialists (obstetricians and pediatricians) working mostly on an on-call basis.

*Years of participation: 2019—present*

#### India (Belagavi)

The research site is within the northwestern corner of the southern state of Karnataka, India, with the site coordinating center located in Belagavi. Each of the clusters corresponds to the service areas of one or more primary health centers. Each is managed by a physician medical officer who works with nursing staff and auxiliary nurse midwives in associated sub-centers, the most peripheral outpost of the health care services. There are three tertiary care hospitals and eight secondary care hospitals serving the region as referral hospitals staffed by obstetricians, pediatricians and nurses. In addition to these public sector health facilities, there are several private sector maternity facilities within the site catchment area.

*Years of participation: 2008—present*

#### India (Nagpur)

The research site is within the state of Maharashtra, India, with the coordinating center based in Nagpur. Each of the clusters corresponds to the service area of 20 primary health centers, and each is served by physician medical officers and nurses. These areas include 117 sub-centers where basic maternal and childcare services are provided. Referral care within the districts of the clusters is provided in ten tertiary hospitals (two in the public sector and eight in the private sector), and 30 secondary hospitals under public sector. In addition to these facilities, there are more than 100 private sector secondary level hospitals and nursing homes.

*Years of participation: 2009—present*

#### Pakistan (Thatta)

Research sites are located in two of five sub-districts within the Thatta district in the southern Sindh province. Sindh is near the city of Karachi, where the site coordinating center is located at Aga Khan University. The study clusters are served by more than 75 health facilities, both public sector and private fee-for-service, providing maternal and child health services. These include 47 primary health clinics, 25 secondary care facilities and 3 referral hospitals. Care in primary health clinics is typically provided by either paramedical staff, including nurses and lady health visitors or non-specialist physicians. Obstetricians and pediatricians provide care in secondary and referral hospitals.

*Years of participation: 2008—present*

### Sub-Saharan Africa sites

#### Kenya

The research site is within the western region of Kenya in the counties of Busia, Bungoma and Kakamega, with the site coordinating center located at Moi University, in Eldoret. The clusters are served by over 20 health facilities, most operated by the government and staffed by nurse-midwives and clinical officers and a single medical officer. Three hospitals in the area function as county referral hospitals. There is a one tertiary teaching and referral hospital based in Eldoret for the western region with a newly established training program in maternal fetal medicine. Most physicians are generalists, with some trained obstetricians and pediatricians.

*Years of participation: 2008—present*

#### Zambia

The MNHR is based south and east of the capital city of Lusaka, Zambia, in four main districts (Kafue, Chilanga, Rufusa and Chongwe) where all the work is conducted. The site coordinating center is located at the University of Zambia, in Lusaka. There are ten clusters, eight of which have health posts. Care is provided primarily by nurses and midwives in the health center and posts and by traditional birth attendants for home births. Currently, there are three district hospitals and two referral hospitals in Lusaka, namely University Teaching Hospital and Levy Mwanawasa Teaching Hospital. Pediatricians and obstetricians are available only in the referral centers.

*Years of participation: 2008—present*

#### Democratic Republic of Congo

The research sites are in the North and South Ubangi Provinces, with the site coordinating center at Kinshasa School of Public Health, in Kinshasa. Each of the study clusters is served by a health center. Care in health centers is provided by nurses. There are two hospitals serving the study catchment area that are staffed by physicians, nurse midwives and nurses; no specialty physicians are available.

*Years of participation: 2013—present*

### Central American site

#### Guatemala

The Chimaltenango region is in the Western Highlands of Guatemala, with the coordinating center based in Guatemala City. The study clusters are served by one referral hospital, 30 health centers, and 42 health posts. Maternal and infant care in the hospital is provided mainly by obstetricians, pediatricians, and general physicians, in health centers by physicians and nurses, and in health posts by auxiliary nurses.

*Years of participation: 2008—present*

### Study population

The objective of the MNHR is to register each pregnant woman residing within the designated communities, also referred to as clusters, and to collect data on these pregnancies and their outcomes. Each cluster is defined by a geographic area including all households within the area. For a cluster to be eligible for the MNHR, generally it needs to be based on a population with approximately 300 to 500 deliveries annually, although the specific numbers may differ. The clusters usually correspond to existing healthcare service delivery areas, such as an area or zone defined by the ministry of health in the participating country. Each site currently has between 8 to 10 active study clusters, but in prior years, some sites have had up to 24 clusters. Altogether, the MNHR enrolls approximately 60,000 pregnant women annually [5].

Pregnant women, and their newborns, who are residents of the study clusters are eligible to participate in the MNHR.

### Enrollment procedures

Study staff created and maintain detailed maps of the health facilities serving each cluster and a log of all providers (e.g. traditional birth attendants) who attend deliveries outside of facilities. A variety of surveillance methods have been utilized to identify pregnant women as early as possible. The study RAs proactively identify women at or prior to antenatal care (ANC) through sensitization activities. In addition, they engage all active birth attendants in the clusters in order to facilitate the documentation of facility as well as home deliveries. On a routine basis, the RAs review hospital and clinic logs for enrollment at ANC as well as for facility births. The study team monitors cluster-level monthly data to identify trends that may indicate missed enrollments. Use of mobile phones is one strategy that has proven effective to facilitate identification and tracking of women at several sites [8]. In addition, sites conduct household surveys to help identify women who are eligible for the MNHR [4].

### Data collection

Data are formally collected at three time-points, at enrollment during pregnancy, within 72 h of delivery and at 42-days post-partum. Additional contacts are made between these formal data collection visits to maintain connection with the pregnant woman and her family. The RA collects data on socio-economic, demographic, health care characteristics and pregnancy outcomes. Standard definitions are used to classify certain outcomes and characteristics. For example, gestational age is estimated using ultrasound, last menstrual period (LMP), or clinical data such as physical examination, and other available information when LMP is unknown. An algorithm, based on recommendations from the American College of Obstetrics and Gynecology, then assigns the gestational age and estimated delivery date for the study [21]. With introduction of ultrasound at many sites, the use of ultrasound-based gestational age has increased over the study period [14]. In addition, the objective is to measure birth weight within 48 h of delivery using weighing scales provided by the study. When birth weight is not obtained, weight is estimated by the RA to distinguish infants weighing less than and greater than 1000 g and 2500 g. Birth attendants are classified as physicians, nurses or equivalent, traditional birth attendants (TBA) or equivalent, family or unattended, while the delivery location is defined as hospital, health center or home (including the TBA’s home or in-transit). Finally, receipt of ANC is defined as having at least one health care visit with a skilled health provider, but the specific number of visits is also documented.

The clinical conditions are recorded by RAs, using the WHO definitions, whenever possible [22]. The major outcomes include stillbirths (fetal demise after 20 weeks gestation and prior to delivery), neonatal death (death at < 28 days), and maternal mortality (death of mother during pregnancy or up to 6-weeks postpartum). These standardized definitions are used to collect the data across the sites, with a manual of operations and training materials used to reinforce the definitions across study sites [9].

The causes of maternal, stillbirth and neonatal deaths are assigned by physicians at each site based on their evaluation of the available clinical information for each case. Prior to 2014, the Global Network did not have a methodology for assigning cause of death systematically across sites, resulting in potential inconsistency across the sites. In 2014, an additional data form was added to collect supplemental data about the deaths and a hierarchal computer-based system to assign cause of death using a prospectively defined methodology was implemented for maternal and neonatal deaths as well as stillbirths [23, 24]. In 2019, a more in-depth socio-economic status data collection tool was added to the MNHR to obtain more granular assessment of the women’s status [25].

### Data management system

Study staff collect all data for women within each cluster; a supervisor then reviews the forms for completeness and accuracy. The computerized data management system also contains basic inter- and intra-form checks. Each site transmits data to the DCC for central analyses and additional data edits to ensure quality [9]. Routine monitoring reports are reviewed at least monthly by each site team to resolve data errors.

### Quality assurance and training

The RAs receive training on the completion of data forms, schedule of data collection and the process for editing data forms [9]. Birth attendants are trained to collect data and assess basic clinical variables and outcomes, including differentiation of stillbirths from early neonatal deaths, birth weight and assessment of gestational age. Birth attendants are also taught to distinguish macerated from fresh stillbirths using pictures depicting levels of maceration.

At each site, the RAs have monthly meetings to review their data collection and have refresher training on study definitions on an annual basis, with specific training held more frequently as needed.

Each site develops a monitoring plan to ensure the quality of the data. The monitoring plan has several components, including a timetable for responding to edits and an assessment of responsibility for completeness of data collection, data quality, data accuracy and data entry [7, 9]. The compliance with the plan is also tracked centrally.

To assist site staff with monitoring activities, the DCC prepares monthly monitoring reports that document trends in study data for key variables. Site-specific programs are also deployed to assist each site in monitoring data locally. Additionally, site visits are conducted routinely by the DCC, NICHD and the core investigator to review the overall study progress as well as the quality of the data collection.

### Ethical approval

The appropriate institutional review boards or ethics research committees of the participating institutions and the ministries of health of the respective countries approve the activities of the MNHR. Initially, approval was sought from the appropriate leader of the participating community. Informed consent for study participation is requested from each pregnant woman (and her partner when available). The Global Network Data Monitoring Committee, appointed by the NICHD, oversees and reviews activities of the MNHR at bi-annual meetings.

## Results

**Fig. 2:**
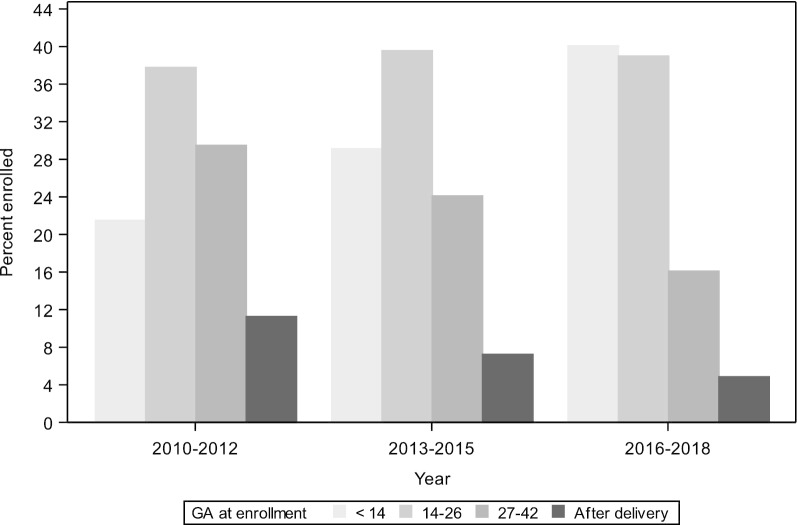
Gestational age at enrollment in the Maternal Newborn Health Registry, by year, 2010–2018

Since the inception of the MNHR in 2008, more than 700,000 pregnant women have been enrolled. During the calendar years 2010–2018, 579,140 pregnant women were enrolled in the MNHR, representing 99.8% of those eligible (Table 2). Of the pregnancies enrolled, delivery data were documented for 99% of women and 42-day follow-up for 99% of women enrolled. The gestational age (GA) at enrollment has varied by site, with the more recent data showing that about 40% of all women are enrolled by < 14 weeks gestation (Fig. 2).

**Table 2:** Enrollment in the Maternal Newborn Health Registry 2010–2018 by study site

Enrollment summary	Overall	DRC	Zambia	Kenya	Guatemala	Belagavi	Nagpur	Pakistan
Screened, N	582,768	32,449	63,415	75,796	85,467	135,481	87,923	102,237
Ineligible, N (%)	2543 (0.4)	0 (0.0)	2 (0.0)	24 (0.0)	72 (0.1)	1 (0.0)	1 (0.0)	2,443 (2.4)
Eligible, N (%)	580,225 (99.6)	32,449 (100)	63,413 (100)	75,772 (100)	85,395 (99.9)	135,480 (100)	87,922 (100)	99,794 (97.6)
Did not consent, N (%)	1085 (0.2)	0 (0.0)	0 (0.0)	3 (0.0)	978 (1.1)	23 (0.0)	0 (0.0)	81 (0.1)
Consented, N (%)	579,140 (99.8)	32,449 (100.0)	63,413 (100)	75,769 (100.0)	84,417 (98.9)	135,457 (100)	87,922 (100)	99,713 (99.9)
Lost prior to delivery, N (%)	5992 (1.0)	523 (1.6)	396 (0.6)	1,554 (2.1)	894 (1.1)	63 (0.0)	373 (0.4)	2,189 (2.2)
Delivered, N (%)	573,148 (99.0)	31,926 (98.4)	63,017 (99.4)	74,215 (97.9)	83,523 (98.9)	135,394 (100)	87,549 (99.6)	97,524 (97.8)
42-day follow-up obtained for mother and baby, N (%)	570,770 (99.6)	31,841 (99.7)	62,619 (99.4)	73,823 (99.5)	83,287 (99.7)	135,319 (99.9)	87,305 (99.7)	96,576 (99.0)

Since the inception of the MNHR, several changes in methodology have been made to improve the quality and accuracy of data collection over the course of the study.

**Fig. 3:**
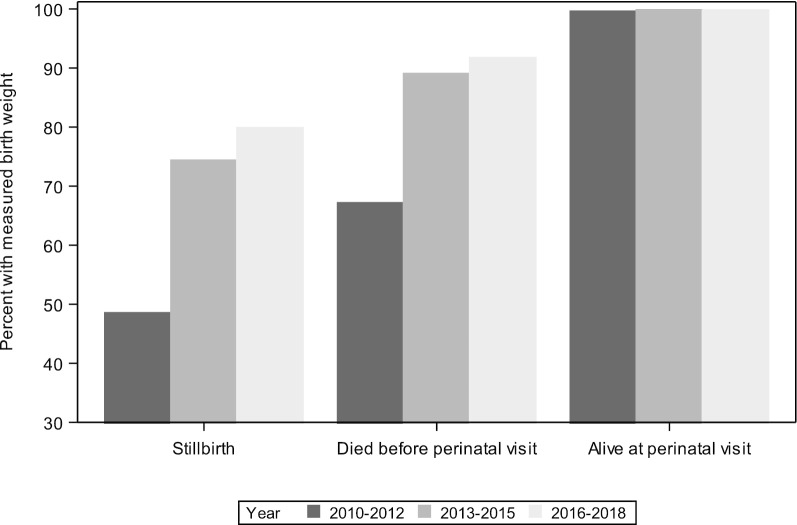
Percent of births with weight measured by fetal/neonatal status at delivery and year of MNHR study, 2010–2018

First, completeness and accuracy of birth weight measurement has improved (Fig. 3). While measurement of the infants born alive has been consistently high (near 98% of all live births), during the 2010–2012 time period, less than half of all stillbirths (48%) were weighed. In the 2016–2018 period, 81% of the stillbirths were weighed. Similar improvements have been made in the measurement of birth weight among early neonatal deaths.

Improvements have also been made in early enrollment of pregnancy (Fig. 2). The initial objective of the MNHR was to enroll all women by 20 weeks gestation. As sites achieved that goal, enrollment at 10–12 weeks became the new target in order to obtain more complete enrollment and to better document early pregnancy loss. Enrollment at early gestational ages now occurs for a substantial proportion of all women, in part due to methods first implemented for other research studies [14–17].

Another improvement that occurred within the MNHR is the increased number of women who receive ultrasound for gestational age dating. The limitations of dating by last menstrual period are well-documented [14, 26]. With advancements in low-cost technology and the implementation of studies in the same catchment areas that utilized ultrasound, gestational age dating using this technology is becoming more common [15].

Finally, women have increasingly sought care in health facilities [10]. As a result of this and other shifts in demographics that have occurred at most sites over time, several key variables, including hemoglobin measurement, are becoming more routine at antenatal care and thus more readily available for MNHR data collection. Data such as these are important as we delve more deeply into factors explaining differences in pregnancy outcomes between study sites.

In addition to the changes in the data collection process, each site has made changes in their study clusters over time for various reasons, including addressing needs of clinical trials (i.e., for a larger number of clusters) or to reduce the size of clusters. When analyses are performed to assess trends over time, the MNHR analyses often include only data from the clusters which continued over the period of interest.

## Discussion

Developing public policy and improving public health in countries with poor perinatal outcomes is, in part, dependent upon understanding the outcome of every pregnancy. Because the worst pregnancy outcomes typically occur in countries with limited health registration systems and vital records, alternative registration systems may be valuable in providing crucial data. One alternative is using a survey system, such as the Demographic and Health Survey (DHS). The DHS has conducted surveys in more than 90 low- and middle-income countries since 1984 [2]. It is widely used for country comparisons but is handicapped in this capacity because it is often adapted by individual countries to suit national needs for specific data.

By contrast, the MNHR has the advantage of using the same data set, data gathering techniques and standard definitions across all sites. The MNHR also is an ideal tool for evaluating the effectiveness of strategies of care because, unlike with the use of periodic surveys, data are collected continuously over time within the same population-based cohort. This strategy enables investigators to determine the impact of interventions to improve outcomes, to monitor trends over time, and to evaluate the changing patterns of perinatal care to inform health policy. In addition to evaluating interventions, the MNHR data have informed a number of global initiatives to understand the maternal, stillbirth and maternal and newborn mortality in LMICs [27–29].

The Global Network MNHR provides prospectively collected, population-based pregnancy outcomes for defined geographic regions within low resource settings. One of the strengths of the MNHR is the population-based nature of the MNHR, which reduces potential bias present in facility-based pregnancy registries. Additionally, because it is a prospective study, it reduces some of the recall bias associated with periodic surveys [2, 30]. Additionally, the MNHR uses standardized definitions and methods across disparate sites that allows for comparisons. Finally, the large annual enrollment allows for increased precision in documentation of relatively rare events, such as maternal mortality.

One of the limitations of the MNHR is the difficulty in ensuring the inclusion of all pregnancies, and especially those with early pregnancy loss. We acknowledge that pregnancies resulting in miscarriages and terminations are currently under-reported. Historically, at most sites, enrollment in the MNHR has been at the time of the first prenatal visit, and this was often after the first trimester. Some sites are now using multiple strategies to encourage earlier initiation of prenatal care. The Belagavi site, for example, screens all women residing within study clusters who are likely to become pregnant in the next year [31]. As part of the health system, these women are offered pregnancy tests and with consent, the results are provided to the MNHR staff. This linkage facilitates most pregnant women being enrolled before the second trimester, which allows for accuracy in analyses of early loss rates.

While we acknowledge potential of missed enrollments, we believe that our enrolled populations approach the vast majority of all women whose pregnancies reach the second trimester, based on comparison at each site with other existing data. For example, in a recent report from the Belagavi site, birth rates reported by the MNHR were higher than projected based on ministry data and other sources, indicating that the surveillance for the MNHR was more comprehensive than the available census data [7]. Some sites encounter challenges in tracking the outcomes of pregnant women who migrate in or out of the study clusters, for example women who travel to the homes of their mothers at the time of delivery. To address these challenges, the MNHR intentionally enrolls all pregnant women identified, regardless of residency and among those who migrate, attempts to obtain minimum data on the pregnancy outcomes via phone or other contact. To facilitate this, numerous systems have been developed and, through monitoring, a relatively stable enrollment rate has been achieved in the affected study clusters.

Some challenges exist in categorizing critical pregnancy outcomes. For example, the proper classification of intrapartum stillbirth versus very early neonatal death and macerated versus non-macerated stillbirth are particularly challenging outcomes [32]. Additionally, determining accurate birth weights of certain groups of infants is difficult. Often the outcomes are challenging to determine due to births occurring outside a facility. For example, obtaining the weight of stillbirths in some communities may not possible because weighing a dead infant is not culturally acceptable. Acquiring an accurate birth weight of a live-born infant delivered at home is often difficult because of the time to confirm a delivery occurred and reach the home and thus it is difficult to weigh the infant within a few days after birth. To overcome this challenge, strategies have included home visitation and providing village chiefs with scales [8]. Despite these issues, over the entire registry we have achieved a measured birth weight for 98.5% of all births in recent years.

## Conclusions

In this supplement, a series of manuscripts describe features of the MNHR and detail key data available from the MNHR since 2010. These include manuscripts that describe the methods to ensure quality of data collection, maternal mortality, neonatal mortality and stillbirth. Several manuscripts also explore potential risk factors in depth. In addition to these specific analyses, the MNHR data have contributed to important global efforts to better understand the rates, trends and causes of stillbirth and maternal and neonatal mortality. The MNHR continues to serve an important role in documenting women’s and newborn health outcomes in low-resource settings.

## Data Availability

Study data will be available through the NICHD Data and Specimen Hub repository (NICHD-DASH).

## Abbreviations

ANC: Antenatal care
DCC: Data coordinating center
DHS: Demographic health surveys
GA: Gestational age
MNHR: Maternal newborn health registry
NICHD: National Institute of Child Health and Human Development
RA: Registry administrators
